# Measurable outcomes of consumer engagement in health research: A scoping review

**DOI:** 10.3389/fpubh.2022.994547

**Published:** 2022-10-17

**Authors:** Emily DeBortoli, H. Peter Soyer, David Milne, Nadeeka Dissanayaka, Coral Gartner, Jeanette Holt, Kym Rae, Laura Robison, Courtney K. Wallingford, Aideen M. McInerney-Leo

**Affiliations:** ^1^Dermatology Research Centre, The University of Queensland Diamantina Institute, The University of Queensland, Brisbane, QLD, Australia; ^2^Dermatology Department, Princess Alexandra Hospital, Brisbane, QLD, Australia; ^3^Human Research Ethics Committee, Translational Research Institute, Brisbane, QLD, Australia; ^4^Faculty of Medicine, Royal Brisbane and Women's Hospital, University of Queensland Centre for Clinical Research, The University of Queensland, Brisbane, QLD, Australia; ^5^School of Psychology, The University of Queensland, Brisbane, QLD, Australia; ^6^Department of Neurology, Royal Brisbane and Women's Hospital, Brisbane, QLD, Australia; ^7^Faculty of Medicine, School of Public Health, NHMRC Centre of Research Excellence on Achieving the Tobacco Endgame, The University of Queensland, Brisbane, QLD, Australia; ^8^The University of Queensland Diamantina Institute, The University of Queensland, Brisbane, QLD, Australia; ^9^Aubigny Place, Mater Research Institute, Brisbane, QLD, Australia; ^10^Faculty of Medicine, University of Queensland, Herston, QLD, Australia; ^11^Australasian Kidney Trials Network, Translational Research Institute, The University of Queensland, Brisbane, QLD, Australia

**Keywords:** consumer, engagement, involvement, healthcare, research, outcomes, community

## Abstract

**Background:**

Consumer engagement is increasingly recognized as an instrumental component of health research, with many institutions and international bodies mandating it as part of the research and funding process. Given an increasing utilization of consumer engagement in health research, it is critical to identify the literature which support its value and tools that capture successful outcomes. To develop an overview of the literature, we conducted an umbrella scoping review exploring important outcomes of consumer engagement in health research combined with a scoping review of relevant frameworks. Specifically, we aimed to capture outcomes which reflect authentic and meaningful consumer engagement.

**Methods:**

Four databases (PubMed, Embase, CINAHL and Cochrane Library) were searched using key search terms. Records were included if they were review articles or frameworks that addressed outcomes of consumer engagement in health research. Data was analyzed using descriptive statistics and an inductive qualitative content analysis. Identified outcomes were sorted based on the three most relevant stakeholder groups (consumer, researcher, institution).

**Results:**

A total of twenty articles that explored a variety of health disciplines were included. We identified fifteen measurable outcomes of consumer engagement in health research. Eight core outcomes were relevant to all stakeholder groups, and were considered fundamental to authentic consumer engagement including (1) trust, (2) empowerment, (3) respect, (4) confidence in the outcomes of the research, (5) transparency of the research process, (6) satisfaction with the consumer engagement program, (7) knowledge and experiences of consumers, and (8) degree of consumer engagement. Outcomes pertaining to specific stakeholder groups included representativeness and diversity of the consumer groups, research relevance to consumers, funding opportunities, quality/validity of the research, recruitment/retention rates, translation and dissemination of research, and interpretation of results.

**Conclusion:**

This review identified key measurable outcomes that could be captured when evaluating the impacts of consumer engagement on health research and the success of consumer engagement programs. All outcomes identified were relatively underexplored within the literature, and inadequately and/or inconsistently evaluated amongst studies. Future research should consult all stakeholder groups to identify outcomes perceived to be reflective of optimal consumer engagement.

## Introduction

Consumer engagement is an evolving topic within health care and is increasingly considered to be an instrumental component of health research (1). The term “consumer” broadly refers to stakeholders who have personal experience with a health condition, including patients, potential patients, careers, and the wider community (2, 3). Consumer engagement in health research refers to the involvement of such stakeholders at various stages of the research process. “Consumer engagement” and “consumer involvement” are used interchangeably in the literature, though there is a growing consensus that the term “involvement” is more indicative of authentic engagement. Given the recency of the utilization of “involvement” this review will use the term ‘engagement' to more accurately reflect the terms used in the historical literature.

Levels of research activity include identifying research priorities, study planning, seeking funding, conducting the research, data interpretation and implementing the findings (4, 5). Consumer engagement involves research carried out with, or by consumers, rather than to, about, or for them (2). Therefore, the aim of authentic consumer engagement is to advance consumers beyond the role of passive research participants and promote them to a position of active contributor and co-partner in the research (6, 7).

The literature articulates proposed benefits of consumer engagement in health research. First, from an ethical standpoint, consumers have a right to be involved in research decisions that may impact their health and well-being, or that of others (2, 3). Second, consumer engagement can increase the transparency and accountability of research organizations and institutions (2, 3, 8). Additionally, consumer engagement can also improve the quality and relevance of research due to the unique perspectives and knowledge that consumers can provide to the research process (5, 6, 9). Finally, those who participate in the research process may also have more confidence in the outcomes of the research, which can enhance the dissemination and implementation of research findings (3, 7).

The increased emphasis on consumer engagement in health research in recent years is reflected in global guidelines and national public health policies (10, 11). Key research organizations in countries such as the United Kingdom (INVOLVE), the United States of America [Patient-Centered Outcomes Research Institute (PCORI)] and Canada (Canadian Institute for Health Research) promote and emphasize the need for consumer engagement in health research (5, 10). In Australia, the National Health and Medical Research Council (NHMRC) in partnership with the Consumers Health Forum, developed a statement on *Consumer and Community Involvement in Health and Medical Research* (12, 13). The statement outlines actionable, pragmatic steps for developing and implementing consumer engagement in health research (7, 12). Some example guidelines for enabling meaningful consumer engagement include planning and implementing consumer engagement as early as possible and reaching out to an appropriately diverse range of consumers (12).

As there is an increased recognition of the importance of consumer engagement in health research, it is essential to identify literature supporting its value (2). Similarly, it would be valuable to identify reporting standards and standardized tools for evaluating consumer engagement to determine program success and also compare the relative success of different approaches (2, 14, 15).

In 2015, Esmail et al. (2) conducted a systematic review to identify outcomes that should be measured when evaluating consumer engagement in health research. The review of records from 2005 to 2013, identified measurable outcomes including impact of consumer engagement on study design, research relevance, quality of the research, and dissemination of findings (2). The use of consumer engagement in health research has, however, expanded considerably since this review in the form of mandates and research policies nationally and internationally (16). Importantly, in 2017 a Guidance for Reporting Involvement of Patients and Public (GRIPP2) standardized tool was subsequently developed (17). The continued expansion of consumer engagement in health research since Esmail et al.'s (2) review may point to new outcomes that are important to measure when evaluating the impact of consumer engagement and success of consumer engagement programs in health research. Therefore, the present study aims to conduct an updated umbrella scoping review to explore the measurable outcomes of consumer engagement and consumer engagement programs in health research from the perspective of the three primary stakeholder groups (consumer, researcher, and institution).

## Methods

We originally attempted to conduct a systematic search of the literature pertaining to methods of evaluating consumer engagement. Systematic reviews offer a comprehensive synthesis and appraisal of original research evidence relevant to a particular research question/s (18). However, due to the broad number of terms ascribed to the topic of consumer engagement and the associated large volume of broadly focused records, this approach proved to be inappropriate. Through this search we also noticed that numerous reviews had been conducted in certain specialties. To overcome such challenges, we refined our approach to conduct a broader scoping review of review articles and frameworks that addressed important outcomes of consumer engagement in health research. However, “involvement” is a relatively new addition and although we included it in our search terms most articles referred to “engagement” rather than “involvement” (see Supplementary Table 1).

An umbrella review is a review of reviews to give a high-level overview (18). Scoping reviews are a way of identifying and mapping the key concepts that underpin a research area (19). Our umbrella and scoping reviews provide an overview of the available literature pertaining to measurable outcomes of consumer engagement in health research and consumer engagement frameworks.

### Consumer engagement

A consumer expert (DM) with experience on human research ethics committees and patient advocate groups was consulted in the writing and reviewal of the manuscript. This approach was developed in accordance with the GRIPP2 short form tool (17) (see Supplementary Table 2). We included a consumer in manuscript writing and critical review to ensure that the highlighted outcomes were of relevance to consumers. The consumer did not receive financial remuneration for their involvement however they were included as an author of the paper.

### Search strategy

Relevant records were identified through a standardized search of PubMed, Embase, CINAHL (Cumulated Index to Nursing and Allied Health Literature) and Cochrane. The searches were filtered by title and abstract to ensure that the records captured were relevant. Articles were restricted to those written in English. Given the relative impact of consumer engagement, no date limits were applied to the literature searches. A combination of search words and Boolean operators “OR” and “AND” were used to address three key search areas (see Supplementary Table 1).

Articles identified from reference and citation (Google Scholar) searches of included articles and/or from field experts were also included. All search results were imported into Covidence, for screening and data extraction (20).

### Inclusion and exclusion criteria

We included reviews and frameworks that referenced outcomes of consumer engagement and consumer engagement programs. This choice was based on keeping the search results manageable, whilst still capturing a complete overview of the subject area and highlighting gaps in the literature. Due to the large volume of primary articles and the fact that reviews on the impact of consumer engagement in health research have been conducted in numerous areas, primary studies were excluded.

### Selection of studies and data extraction

To assess eligibility for inclusion in the study, the titles and abstracts of all articles resulting from the literature search were independently reviewed by two authors, ED and CW. Authors ED, CW, and AM-L then independently reviewed the full text of the selected articles. At both stages, inconsistencies were addressed and consolidated between authors. Following the filtering of studies, selected articles were summarized in tabular form, which included bibliographic details; study design; key findings; outcomes of consumer engagement or consumer engagement programs; relevant stakeholders; and any frameworks or evaluation tools referenced.

### Data analysis

The data was analyzed using descriptive statistics and through descriptive qualitative content analysis using an inductive approach (21). Researchers ED and CW created a basic codebook and conducted the content analysis manually. Final measurable outcomes of consumer engagement in health research were mapped according to the most relevant stakeholder groups (consumer, researcher, institution).

## Results

**Figure 1:**
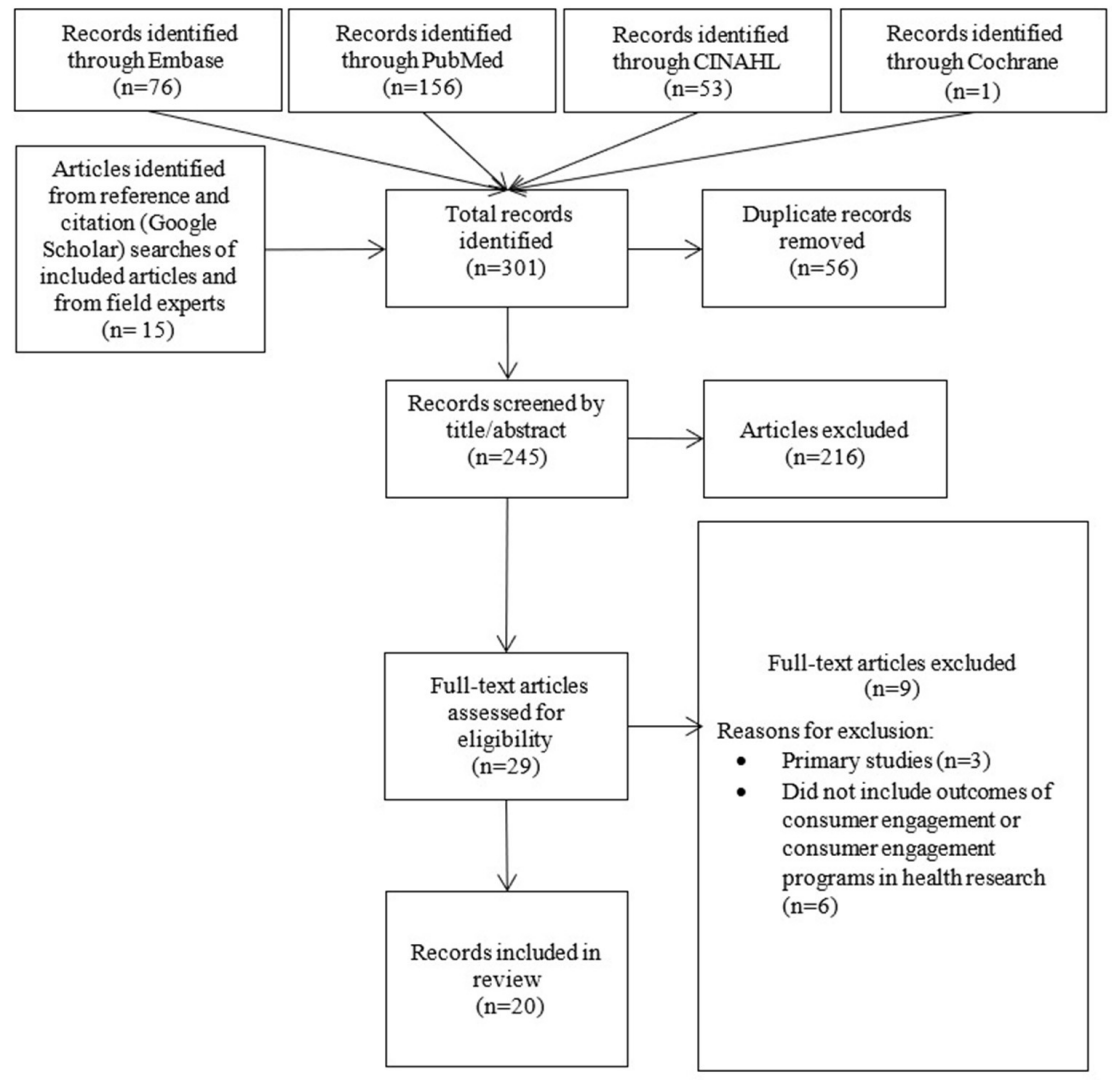
PRISMA flowchart of database and citation research.

A total of 301 records were identified from the initial database searches, and from reference and citation searches of included articles and field experts. Figure 1 depicts a PRISMA flowchart of the database and citation searches. Following the removal of 56 duplicates, the titles, and abstracts of 245 records were screened. A total of 216 records were excluded at this stage as they did not meet the inclusion criteria. The remaining 29 records underwent full text screening, and a further nine were excluded as they did not detail outcomes of consumer engagement in health research and/or because they were primary studies. This left a total of twenty eligible records for data extraction (see Supplementary Table 3).

Specific health research settings where consumer engagement had been considered or measured included public health, comparative effectiveness research, and Indigenous health care. Most records however pertained to primary health care settings. Publication dates ranged from 2005 to 2021, with the majority (*n* = 12/20) of records published since 2015, highlighting the relevance of this review. Over half of the records were published in the United States (*n* = 6/20) and Canada (*n* = 5/20). Of the remaining nine records, four were published in the United Kingdom, two were published in Australia, two in Norway and one in the Netherlands. Three-quarters (*n* = 15/20) of the records were reviews of consumer engagement in health research and almost half of the records (*n* = 9/20) outlined a framework which addressed important outcomes for consideration. Additionally, the majority (*n* = 16/20) of the records denoted the use of consumer engagement as part of the research process (see Supplementary Table 3). However, most did not clarify whether consumers were included as co-authors.

**Figure 2:**
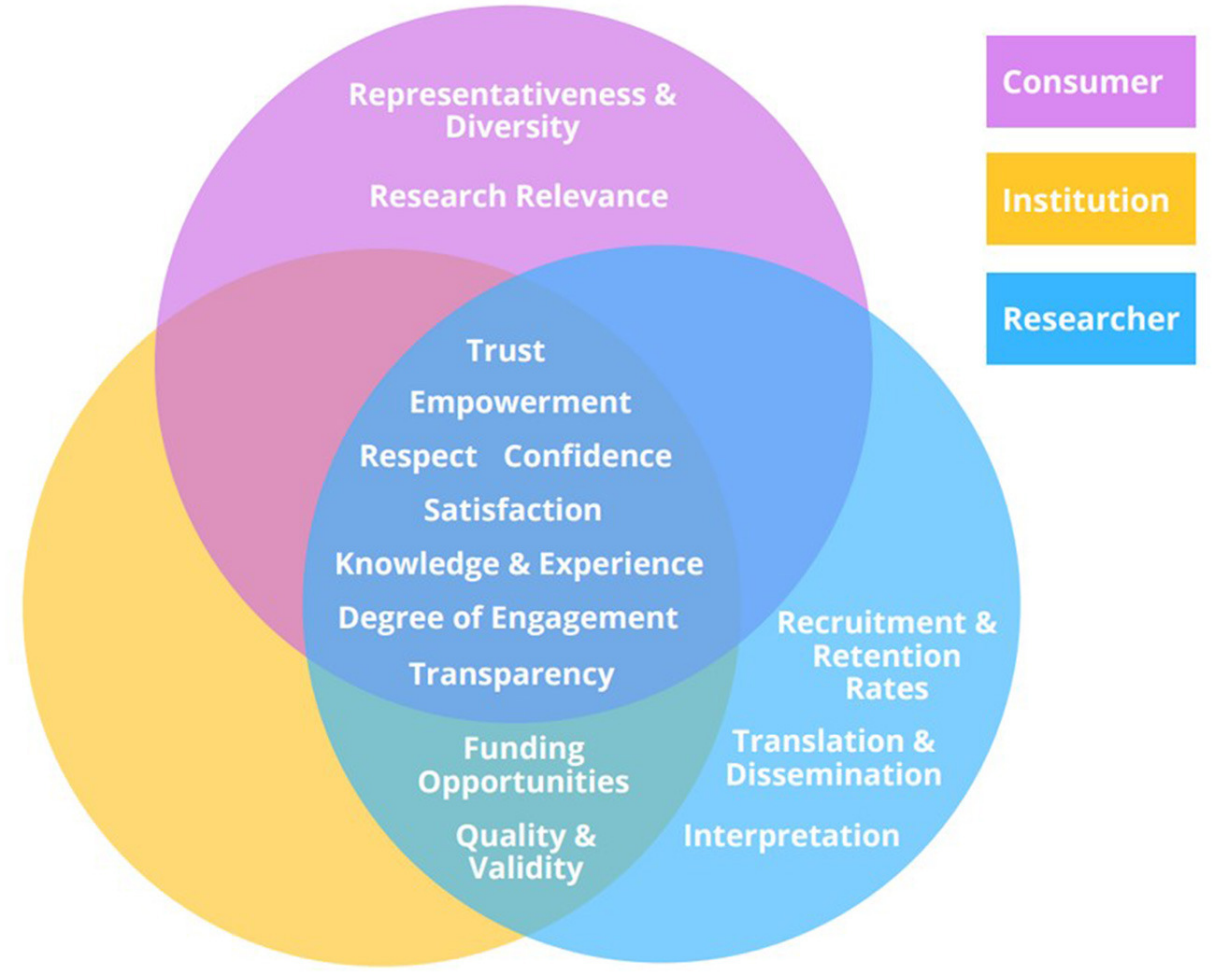
Measurable outcomes of consumer engagement mapped to relevant stakeholders.

Through the review of the literature, we identified fifteen measurable outcomes of consumer engagement and consumer engagement programs in health research. Eight care outcomes were considered fundamental to authentic consumer engagement as they were relevant to all stakeholders. Five of the eight core outcomes reflect the impact of consumer engagement on the research: (1) trust in the institution, (2) empowerment, (3) respect, (4) confidence in the outcomes of the research, and (5) transparency of the research process. Three of the eight core outcomes were relevant to the success of consumer engagement programs: (6) satisfaction with the consumer engagement program, (7) knowledge and experiences, and (8) degree of consumer engagement. Additional outcomes which pertained to specific stakeholder groups included representativeness and diversity of the consumer groups, and research relevance (for the consumer group), funding opportunities, and quality/validity of the research (for the researcher and institution), and recruitment/retention rates, translation and dissemination of research, and interpretation of results (for researchers). Table 1 summarizes all outcomes, the frequency with which they were reported and the corresponding articles. Underreported and/or underexplored outcomes, mentioned within four or fewer records, were highlighted. Figure 2 maps all the identified outcomes and how they relate to each stakeholder.

**Table 1:** Measurable outcomes of consumer engagement categorized relevant to stakeholders.

**Stakeholder**	**Outcomes**	**Number of records which identified outcome**
Consumer	Representativeness and diversity of consumers engaged (2, 21, 23)*	*n* = 3/20
	Research relevance to consumers (2, 8, 16)*	*n* = 3/20
	Respect amongst stakeholders (9, 23, 25)*	*n* = 3/20
	Trust amongst stakeholders (2, 16, 21–24)	*n* = 6/20
	Transparency of research activities (1, 2, 8, 21)*	*n* = 4/20
	Confidence in the consumer engagement program (3, 16)*	*n* = 2/20
	Satisfaction with the consumer engagement program (1, 2, 16, 27)*	*n* = 4/20
	Empowerment (10, 16, 27)*	*n* = 3/20
	Knowledge and experience (2, 3, 16, 22, 26, 27, 32)	*n* = 7/20
	Degree of consumer engagement in research (2, 3, 8, 12, 21–23, 25)	*n* = 8/20
Researcher	Translation and dissemination of research findings (2, 8, 9, 12, 22, 26–30)	*n* = 10/20
	Interpretation of results (2, 8, 29, 30)*	*n* = 4/20
	Recruitment and retention rates of participants (2, 9, 25, 27, 28)	*n* = 5/20
	Quality and validity of research (2, 27)*	*n* = 2/20
	Funding opportunities (3, 31)*	*n* = 2/20
	Respect amongst stakeholders (9, 23, 25)*	*n* = 3/20
	Trust amongst stakeholders (2, 16, 21–24)	*n* = 6/20
	Transparency of research activities (1, 2, 8, 21)*	*n* = 4/20
	Confidence in the consumer engagement program (3, 16)*	*n* = 2/20
	Satisfaction with the consumer engagement program (1, 2, 16, 27)*	*n* = 4/20
	Empowerment (10, 16, 27)*	*n* = 3/20
	Knowledge and experience (2, 3, 16, 22, 26, 27, 32)	*n* = 7/20
	Degree of consumer engagement in research (2, 3, 8, 12, 21–23, 25)	*n* = 8/20
Institution	Quality and validity of institution (2, 27)*	*n* = 2/20
	Funding opportunities (3, 31)*	*n* = 2/20
	Respect amongst stakeholders (9, 23, 25)*	*n* = 3/20
	Trust amongst stakeholders (2, 16, 21–24)	*n* = 6/20
	Transparency of research activities (1, 2, 8, 21)*	*n* = 4/20
	Confidence in the consumer engagement program (3, 16)*	*n* = 2/20
	Satisfaction with the consumer engagement program (1, 2, 16, 27)*	*n* = 4/20
	Empowerment (10, 16, 27)*	*n* = 3/20
	Knowledge and experience (2, 3, 16, 22, 26, 27, 32)	*n* = 7/20
	Degree of consumer engagement in research (2, 3, 8, 12, 21–23, 25)	*n* = 8/20

* Underreported and/or underexplored outcomes are those identified in four or fewer records.

The literature we reviewed suggests that consumer engagement in health research can lead to the inclusion of more diverse and representative viewpoints (2, 22, 23) and elicit research that is more relevant to consumers from a broad range of demographic backgrounds (2, 8, 16). Ten studies found that consumer engagement in health research enabled greater ease of translation and dissemination of research findings (2, 8, 9, 12, 24–29) and more insightful interpretation of findings (2, 8, 28, 29). Some studies detailed that consumer engagement improved the overall quality and validity of the research (2, 26), and increased the number of funding opportunities (3, 30). Five studies reported that consumer engagement improved recruitment and retention rates of participants (2, 9, 23, 26, 27). Successful consumer engagement in health research promotes high levels of confidence in the research and institution (3, 16) and satisfaction regarding the consumer engagement program amongst all stakeholders (1, 2, 16, 26). Consumer engagement may facilitate greater transparency of the research process by increasing stakeholder's accountability (1, 2, 8, 22) and enhance the level of trust in (2, 16, 22, 24, 31, 32) and respect for all stakeholders (9, 23, 31). Early and sustained consumer engagement increases the action ability of findings and ensures that consumer perspectives are captured and optimized across the research process (2, 3, 8, 12, 22–24, 31). Consumer engagement also results in improved knowledge and understanding of the research topic (2, 3, 16, 24–26, 33) and feelings of empowerment amongst all stakeholders (10, 16, 26).

## Discussion

Our scoping review identified fifteen measurable outcomes of consumer engagement and consumer engagement programs in health research, as relevant to three primary stakeholder groups (consumer, researcher, and institution). Eight core outcomes were considered key to authentic consumer engagement, as they were relevant to all stakeholder groups. Core outcomes included those that reflected the impacts of consumer engagement on research (trust, empowerment, respect, confidence in the outcomes of the research, and transparency of the research process) and those relevant to the success of consumer engagement programs (satisfaction with the consumer engagement program, knowledge and experiences of consumers, and degree of consumer engagement). Equally important outcomes which pertained to specific stakeholder groups included representativeness and diversity of the consumer groups, as well as research relevance (for the consumer group), funding opportunities, and quality/validity of the research (for the researcher and institution), and recruitment/retention rates, translation and dissemination of research, and interpretation of results (for researchers). Importantly, all identified outcomes were relatively underexplored within the reviewed literature and were inadequately and/or inconsistently evaluated amongst studies.

Of note the lack of literature exploring the representativeness and diversity of consumers engaged in health research is concerning, as engaging consumers who are reflective of the wider population group is integral to ensuring that research promotes equitable health outcomes (2, 22, 23). Similarly, research relevance to consumers is underexplored within the literature. Consumer engagement in health research aims to improve the relevance of the research to consumers and ensure that it reflects their needs (2, 16, 19). Therefore, to mitigate the risk of tokenistic consumer engagement, it is important to measure the relevance of the research to consumers as an outcome. Additionally, the limited literature citing stakeholders' levels of confidence and satisfaction with consumer engagement programs raises concerns as higher levels of stakeholder confidence and satisfaction facilitate improved dissemination and implementation of findings (2, 3, 26). Such metrics are also considered to improve retention rates, predict future consumer engagement, and promote public trust in research (16).

Despite increasing research in this field and the recent development of a standardized tool for reporting on the broad outcomes of consumer engagement in health research (17), we only identified two tools in the reviewed literature which evaluated the outcomes of consumer engagement and consumer engagement programs in health research. While not yet standardized, a tool developed by Vat et al. (16) attempts to monitor and evaluate the impact of consumer engagement in health research by measuring outcomes such as, the transparency of research activities, research relevance, quality and validity of the research conducted, and representativeness and diversity of engaged consumer through surveys, interviews, questionnaires, and reflection session. Similarly, Concannon et al. (8) developed a seven-item questionnaire that evaluated the impact of consumer engagement in health research by measuring outcomes including, representativeness and diversity of consumers engaged, research relevance, transparency of research activities, and dissemination of findings. Neither the former or latter tool captured perspectives of stakeholders beyond the consumers or institutional considerations. Although not all specific to health research, four additional tools have been identified in the broader scientific and gray literature that could be adapted for use within health research to evaluate knowledge and experience, confidence, relevance, and recruitment/retention rates (21, 34–36).

In general, in lieu of standardized evaluation tools, outcomes of consumer engagement in health research are largely reported anecdotally (2). Some outcomes i.e. personal experiences, might be more comprehensively or sensitively captured using a mixed methods or qualitative methodology. The implementation of qualitative methodologies would ensure a more holistic approach and mitigate the risk of anecdotal reports. Inconsistent methods of evaluating and substantiating the value of consumer engagement in health research in conjunction with mandates for consumer engagement could lead to less meaningful and effective engagement strategies. This concern of tokenistic consumer engagement is echoed in the literature and is juxtaposed with authentic, meaningful consumer engagement (25, 32). It is suggested that tokenistic consumer engagement results from inauthentic intentions and a lack of understanding and practice of engagement (32). Encouragingly, of the twenty articles included in this review, sixteen reported consumer engagement, involvement, or consultation. Only one specified that a consumer had been included as a co-author. It is unknown whether the remaining fifteen did not include a consumer as a co-author or whether they omitted to document their inclusion. However, if it is the former, then this is an important consideration for future research. This review identified several outcomes pertaining to the success of consumer engagement programs, including stakeholders' knowledge and experience, degree of engagement, and levels of satisfaction and confidence with the program, alongside the relevance of the research to consumers, and representativeness and diversity of consumer engaged. The development of new consumer engagement programs should consider how best to enhance each measurable outcome pertaining to the success of consumer engagement programs. This will ensure the potential benefits of consumer engagement on research outcomes and consumer experience are realized. Regarding stakeholders' knowledge and experience, previous literature has shown that adequate stakeholder support through training, education, and resources is fundamental to successful consumer engagement programs (10, 23, 30). Sufficient resource availability and funding may also enhance the level of research relevance to consumers (2, 10, 16). In relation to the degree of engagement, it is suggested that early and sustained consumer engagement across research activities facilitates successful consumer engagement programs (31). It is also imperative that consumer engagement programs are managed appropriately and that there are opportunities for stakeholders to provide feedback on outcomes such as their level of satisfaction and confidence with the program (16). Finally, for representativeness and diversity of consumers, there ought to be an overarching policy that guides consumer engagement programs, in conjunction with a consumer engagement coordinator/officer who advocates for, liaises with, and mandates the use of consumer engagement (16).

## Future implications

In order to substantiate the value of consumer engagement in health research future studies should focus on the key outcomes identified in this review and how they would be most effectively captured and evaluated. The development of standardized, validated, and co-designed tools for evaluating outcomes of consumer engagement is critical to demonstrate the authenticity, effectiveness and impact of engagement programs. There needs to be consensus on which factors may be more comprehensively evaluated using a qualitative or mixed methods approach. When creating or developing new consumer engagement programs, it is crucial to identify the desired outcomes and indicators of a success before deciding how they can be most effectively captured.

## Strengths and limitations

This study offers a map of the existing literature and synthesizes key measurable outcomes that are important to consider when evaluating the impact of consumer engagement in health research. This review captured a broad range of papers that explored a variety of health disciplines, from Europe, North America and Australia, and the generalizability is limited accordingly. The lack of literature pertaining to Asia, Africa, and South America highlights the need to evaluate the desired outcomes of consumer engagement across other countries and cultures and consider the importance of cultural influences. This review also offers a direction for future research and outlines gaps surrounding the development and employment of standardized tools to evaluate the impact of consumer engagement in health research and the success of consumer engagement programs. Limitations for this review include that multiple terms encompass ‘consumer engagement' and thus some papers may have been missed due to alternate keywords. A further limitation is that primary research articles were excluded, as is typical in umbrella and scoping reviews.

### Consumer engagement

Involving a consumer in the reviewal of the manuscript provided us with a more comprehensive understanding about how the findings could be interpreted. We acknowledge that whilst a consumer was involved in the writing and reviewing of the manuscript, including them in all stages of the research process would have been more valuable. Furthermore, including more than one consumer would have added further richness to the interpretation.

## Conclusion

This scoping review highlighted key measurable outcomes of consumer engagement in health research that could be considered when evaluating the impact of consumer engagement in health research and success of consumer engagement programs. We identified eight core outcomes relevant to all stakeholders, and seven pertaining to specific stakeholder groups, considered fundamental to authentic consumer engagement in health research. While the majority were underreported, more focus should be placed on evaluating representativeness and diversity of consumer groups, research relevance to consumers, stakeholder confidence and satisfaction with the consumer engagement program. Historically, consumer engagement outcomes have been selected based on assumptions. It may be valuable to consult all stakeholder groups to agnostically elicit the desired outcomes, which they perceive are indicative of optimal consumer engagement.

## Data availability statement

The original contributions presented in the study are included in the article/Supplementary material, further inquiries can be directed to the corresponding author/s.

## Author contributions

ED: conceptualization, formal analysis, methodology, visualization, writing—original draft, and writing—review and editing. HS: supervision, conceptualization, and writing—review and editing. DM, ND, CG, JH, KR, and LR: writing—review and editing. CW and AM-L: supervision, conceptualization, formal analysis, methodology, visualization, writing—original draft, and writing—review and editing. All authors contributed to the article and approved the submitted version.

## Funding

HS holds an NHMRC MRFF Next Generation Clinical Researchers Program Practitioner Fellowship (APP1137127). Funding support by Mater Foundation and Equity Trustees (ANZ QLD Community Foundation, QCF-ANZ Bank Fund, QCF-Thomas George Swallow Trust, The HJ Hinchey Cht Trust). AM-L was funded by a National Health and Medical Research Council (NHMRC) Early Career Fellowship (ID 1158111). CW was supported by an Australian Government Research Training Program Scholarship.

## Conflict of interest

Author HS was a shareholder of MoleMap NZ Limited and e-derm consult GmbH and undertakes regular teledermatological reporting for both companies and he was a Medical Consultant for Canfield Scientific Inc, MoleMap Australia Pty Ltd, Blaze Bioscience Inc, and a Medical Advisor for First Derm. The remaining authors declare that the research was conducted in the absence of any commercial or financial relationships that could be construed as a potential conflict of interest.

## Publisher's note

All claims expressed in this article are solely those of the authors and do not necessarily represent those of their affiliated organizations, or those of the publisher, the editors and the reviewers. Any product that may be evaluated in this article, or claim that may be made by its manufacturer, is not guaranteed or endorsed by the publisher.
