# Supplementary material for: Measurable outcomes of consumer engagement in health research: A scoping review
Source: Front Public Health. 2022 Oct 17;10:994547. doi: 10.3389/fpubh.2022.994547 (PMC9621387; doi:10.3389/fpubh.2022.994547)
Supplement: Supplementary file 1 [file Data_Sheet_1.docx]

**Supplementary Material**

**Supplementary Table 1: Search Terms**

| **Search area** | **Search terms** |
| --- | --- |
| What | “Consumer engagement” OR  “Consumer involvement” OR  “Community involvement” |
| Setting | “Health care” OR  Health AND  Research |
| Study type | Framework OR  Review |

**Supplementary Table 2: GRIPP2 Short Form (17)**

| **Section &**  **topic** | **Item** | **Reported** | **Consumer feedback** |
| --- | --- | --- | --- |
| Aim | Report the aim of PPI in the study |  | In agreeance |
| Methods | Provide a clear description of the methods used for PPI in the study |  | In agreeance |
| Study results | Outcomes – report the results of PPI in the study, including both positive and negative outcomes |  | “A *key finding*” (PPI can lead to more diverse and representative viewpoint)  “*A key benefit*” (PPI improved recruitment and retention rates of participants) |
| Discussion & conclusion | Outcomes – comment on the extent to which PPI influenced the study overall. Describe positive and negative effects |  | “*Tokenism is all too prevalent. Ticking the box because consumer input is mandated. This merits a further comment*” |
| Reflections /critical perspective | Comment critically on the study, reflecting on the things that went well and those that did not, so others can learn from this experience |  | [*…consider that not all outcomes will be best measured using quantitative methods. Qualitative analysis can give use a rich insight into the effectiveness of consumer engagement]* |

PPI=patient and public involvement

**Supplementary Table 3: Records Included in Review**

| **Reference** | **Consumer engagement noted** | **Consumer authorship noted** |
| --- | --- | --- |
| Nilsen et al. (1) |  |  |
| Esmail et al. (2) |  |  |
| Deverka et al. (3) |  |  |
| Concannon et al. (8) |  |  |
| Domecq et al. (9) |  |  |
| Brunton et al. (10) |  |  |
| Gunatillake et al. (12) |  |  |
| Vat et al. (16) |  |  |
| Greenhalgh et al. (21) |  |  |
| Lavery et al. (22) |  |  |
| Manafo et al. (23) |  |  |
| Snape et al. (24) |  |  |
| Duffett et al. (25) |  |  |
| Hamilton et al. (26) |  |  |
| Harris et al. (27) |  |  |
| Oldfield et al. (28) |  |  |
| Lin et al. (29) |  |  |
| Shippee et al. (30) |  |  |
| Miller et al. (31) |  |  |
| Minogue et al. (32) |  |  |

**=** noted
